# Effect of Tai Chi on Young Adults with Subthreshold Depression via a Stress–Reward Complex: A Randomized Controlled Trial

**DOI:** 10.1186/s40798-023-00637-w

**Published:** 2023-09-28

**Authors:** Jingsong Wu, Jian Song, Youze He, Zhaoying Li, Haiyin Deng, Zhenming Huang, Xiaoting Xie, Nichol M. L. Wong, Jing Tao, Tatia M. C. Lee, Chetwyn C. H. Chan

**Affiliations:** 1 College of Rehabilitation Medicine, Fujian University of Traditional Chinese Medicine, Fuzhou, People’s Republic of China; 2 The Academy of Rehabilitation Industry, Fujian University of Traditional Chinese Medicine, Fuzhou, People’s Republic of China; 3 State Key Laboratory of Brain and Cognitive Sciences, The University of Hong Kong, Rm 656, The Jockey Club Tower, Pokfulam Road, Pokfulam, Hong Kong People’s Republic of China; 4 Laboratory of Neuropsychology and Human Neuroscience, The University of Hong Kong, Pokfulam, Hong Kong People’s Republic of China; 5 National-Local Joint Engineering Research Center of Rehabilitation Medicine Technology, Fujian University of Traditional Chinese Medicine, Fuzhou, People’s Republic of China; 6 Fujian Key Laboratory of Rehabilitation Technology, Fujian University of Traditional Chinese Medicine, 1 Huatuo Road, Minhou Shangjie, Fuzhou, 350122 Fujian People’s Republic of China; 7 Department of Psychology, The Education University of Hong Kong, Tai Po, Hong Kong People’s Republic of China

**Keywords:** Tai Chi training, Subthreshold depression, Salivary cortisol level, Gray matter volume, Randomized controlled trial

## Abstract

**Background:**

Subthreshold depression is a highly prevalent mood disorder in young adults. Mind–body exercises, such as Tai Chi, have been adopted as interventions for clinical depressive symptoms. However, the possible effect and underlying mechanism of Tai Chi on subthreshold depression of young individuals remain unclear. This randomized controlled study aimed to evaluate the effects of Tai Chi training and tested the combined stress and reward circuitry model for subthreshold depression.

**Results:**

A total of 103 participants completed this trial, with 49 in the 12-week 24-style Tai Chi group and 54 participants in control group. Our results showed significantly lower scores on depressive symptoms (*P* = 0.002) and anxiety symptoms (*P* = 0.009) and higher scores on quality of life (*P* = 0.002) after Tai Chi training. There were significant reductions in salivary cortisol levels (*P* = 0.007) and putamen gray matter volume (*P* < 0.001) in the Tai Chi group. The changes in cortisol levels and putamen gray matter volume had direct (bootstrapping confidence interval [− 0.91, − 0.11]) and indirect effects (bootstrapping confidence interval [− 0.65, − 0.19]) on the changes induced by Tai Chi training on depressive symptoms, respectively.

**Conclusion:**

The stress–reward complex results indicated an interaction between lowering stress levels and increasing reward circuitry activity associated with the alleviation of depressive symptoms among participants. The 12-week Tai Chi training was effective in improving the symptoms and quality of life of young adults with subthreshold depression.

*Trial Registration* Chinese Registry of Clinical Trials (Registration Number: ChiCTR1900028289, Registered December 12, 2019).

**Supplementary Information:**

The online version contains supplementary material available at 10.1186/s40798-023-00637-w.

## Key points

- Tai Chi training improves depressive and anxiety symptoms and quality of life in young people with subthreshold depression.
- Improvements in depressive symptoms are associated with reduction in putamen volume and salivary cortisol levels.
- Involvement of the stress and reward circuit suggests Tai Chi would improve stress regulation and reward preference.

**Supplementary Information:**

The online version contains supplementary material available at 10.1186/s40798-023-00637-w.

## Background

Major depressive disorder (MDD) is a global public health issue of growing concern [1]. According to the Diagnostic and Statistical Manual of Mental Disorders (DSM-V), MDD is a progressive disorder [2]. Subthreshold depression is defined as having at least two depressive symptoms, but not meeting the MDD criteria [3]. The prevalence of subthreshold depression among young adults aged 18–24 years (14.3%) was found to be higher than that in the general population (11.0%) [4, 5], with a 10–20% risk of developing MDD in later life [3].

Stress and reward are two common constructs to understand the onset of depression. Three physiological mechanisms have been proposed to explain the stress construct. They are the low level of brain-derived neurotrophic factor, glutamic acid-mediated toxicity, and hypothalamus–pituitary–adrenal (HPA) axis dysfunction [6, 7]. The neurobiological- and pathophysiological-related mechanisms, however, do not articulate the processes related to individuals’ emotional processing and responses that are crucial elements in the diagnosis of MDD. In contrast, the dysfunction of the HPA axis has been associated with individuals’ failure in reward expectation and regulation of the associated emotional behaviors [8]. The free cortisol levels were found to increase in patients with depression [9, 10]. The elevated cortisol level was reported to be associated with the impaired structural integrity of the reward circuit [11, 12], which, in turn, reduced the activity of reward expectation [13] and reward response [14]. These behavioral changes align closely with the criteria for diagnosing MDD in the DSM-5. Henceforth, we adopted both the HPA axis activity and reward circuitry as the theoretical basis for the current study.

The reward processes such as motivation, reinforcement learning, and hedonic ability [15] are closely related to the pleasure deficit and lack of motivation defined as depressive symptoms. The reward processes involve extensive brain regions including the prefrontal cortex [16], striatum [17], globus pallidus [18], amygdala [12], insula [14], and hippocampus and thalamus [19] which form the reward circuitry [20]. The striatum receives dopaminergic input from neurons in the ventral tegmental area and extends to the cerebral cortex [16]. The striatum plays a major role within the reward circuitry to subserve processing of the information [21, 22] and regulates emotional responses associated with the reward [14, 22]. High-risk individuals with depression showed lowered striatal activities in response to reward expectations [13]. Individuals with depression and anhedonia were found to have altered functional connectivity within the ventral striatum [23, 24].

The hypothalamic–pituitary–adrenal (HPA) axis subserves stress adaptation, and its dysfunction is associated with chronic stress [25]. Chronic stress is a primary contributing factor to depression. The previous studies suggested that cortisol, a steroidal hormone, is the marker of the HPA axis activity [26, 27]. Cortisol binds to receptors located at the prefrontal cortex [28], striatum [29], and hippocampus [30] subserving an individual’s emotional recognition [31] and regulation [32]. Patients diagnosed with subthreshold depression or MDD showed an elevated level of free cortisol [9, 10], while their remissions showed a lower level [33].

Studies have found that levels of chronic cortisol release have been associated with rewarding behavior [34] and activation of reward-related brain regions [21, 35]. Increased cortisol concentrations are associated with structural alterations in the reward circuitry [36]. The prefrontal cortex and limbic regions in the reward circuit are also important regulators of the HPA axis activity [37], and elevated cortisol levels in depressed patients may compromise the structural integrity of the reward circuit [11, 12]. Depressive patients were found to be striatal hypersensitized during stress, which manifested by the enhanced activation of the caudate nucleus, nucleus accumbens, and putamen, and were closely related to cortisol secretion [38]. Given the close relationship revealed among depressive patients, combining the HPA axis with the reward circuitry would help characterize the development of depressive symptoms [29]. No study has adopted this combined model to explore the changes among individuals with subthreshold depression in response to mind–body interventions such as Tai Chi training.

Mind–body exercises, such as yoga [39], mindfulness [40], and Tai Chi [41], have been adopted as interventions for individuals with MDD. Among these interventions, Tai Chi training has been extensively tested for its effects on alleviating anxiety and depressive symptoms in young college students [42] and middle-aged people [43]. The mechanism of Tai Chi training was suggested to strengthen the functional connectivity of the fronto-striatal network, particularly between the orbitofrontal gyrus and caudate nucleus [44]. Besides, a 12-week Tai Chi training was found to improve the depressive symptoms of older adults which was associated with increased functional connectivity of the default mode network (DMN) [45]. A recent study expanded the DMN to include the caudate [46]. Our research team’s work on Tai Chi training among a group of healthy older adults revealed both increases in the functional connectivity between the medial frontal cortex and right putamen/caudate [47] and increases in the gray matter volume (GMV) in the insular, medial temporal lobe, and putamen [47, 48]. The above evidence prompted us to, among the extensive neural substrates related to the HPA axis activity and reward circuitry, place emphasis on the caudate and the striatum as the main neural markers for testing the effects of the Tai Chi training.

Based on the above, we hypothesized that 12-week Tai Chi training would induce positive changes in depressive symptoms in participants with subthreshold depression. Positive behavioral changes may be associated with a reduction in the salivary cortisol levels, indicating a lowering of the HPA axis activity. At the same time, positive behavioral changes would be associated with changes in the GMV in neural substrates within the reward circuitry. Finally, we also hypothesized that the HPA axis activity and reward circuit may play different roles in the development of subthreshold depression.

## Methods

### Participants

Young adults were recruited from local communities in a city in China. The inclusion criteria were as follows: (1) young adults between 18 and 25 years [49, 50]; (2) score ≥ 16 on the Centre for Epidemiological Studies Depression Scale (CES-D) [51]; (3) no clinical intervention for depressive symptoms in the past 6 months; (4) did not practice regular mind–body exercise in the past 6 months; and (5) no contraindication to MRI examination. An exclusion criterion was the presence of suicide tendency as measured by the suicidality scale (cutoff score ≥ 6) [52] of the Mini International Neuropsychiatric Interview (MINI version 5.0). The withdrawal criteria for the participants were: (1) volunteering to withdraw; (2) developing serious diseases that would not allow continuation of the intervention; (3) suffering from serious adverse events related to the intervention; and (4) receiving Tai Chi training during the waitlist period. All participants received detailed information on the study, and their written informed consent was obtained.

### Study Design and Procedure

The single-blind randomized controlled trial was conducted from December 2019 to December 2021. Eligible participants were randomly assigned in a 1:1 ratio to the Tai Chi or waitlist control group using a simple allocation sequence generated using IBM Statistical Product Service Solutions (SPSS) v24.0 software. The group allocation was conducted by a project assistant working on the study. Two experienced clinicians who were not involved in the group assignment and delivery of the interventions administered the outcome measures. This study was registered in the Chinese Registry of Clinical Trials (Registration Number: ChiCTR1900028289, Registered December 12, 2019). The trial study protocol was published elsewhere [53].

### Interventions

The Tai Chi training required the participants to practice the 24 simplified styles that were recommended by the General Administration of Sports of China [54]. The training program was 12 weeks in duration; and its content is described in detail in Additional file 1: Table S1. The training was led by a Tai Chi master who had more than 5 years of conducting Tai Chi training classes. There were 36 sessions in total (each 60 min; three times a week), and all sessions were conducted in a gymnasium. Each session followed the same sequence of a 10-min warm-up, a 45-min Tai Chi practice, and a 5-min cool-down. The Tai Chi master instructed the participants on the postures and movements specific to the 24 styles in three sessions during the 1st week. From weeks 2 to 12, the participants practiced three to four cycles of the 24 styles depending on the mastery levels of the participants in each session. The same Tai Chi master covered all the sessions and monitored the performance of the participants.

The control condition was a waitlist for 12 weeks. Participants were asked to maintain their lifestyle and daily activities as usual. However, they were instructed and reminded to refrain from participating in regular physical activity particularly mind–body activity including Tai Chi. By the end of the 12-week waitlist, participants in the control group were given the option to join the Tai Chi training.

Both the Tai Chi and waitlist groups were required to maintain a daily activity log to record daily physical activities and submit it to the researchers by the end of the 12th week. The information on the participants’ daily activities was collated for comparison (Supplementary Material—Table 2).

### Primary Outcome

The PHQ-9 measures the severity of the participants' depressive symptoms [55]. It has nine items, scored from 0 to 3, for participants to recall and rate the frequency of the symptoms experienced in their daily lives. The total score of PHQ-9 ranges from 0 to 27, with higher scores suggesting more frequent depressive symptoms. The Chinese version PHQ-9 was validated and showed satisfactory validity and reliability (Cronbach’s *α* = 0.86) [56]. The baseline and post-intervention PHQ-9 were administered 1 week before commencement and 1 week after completion of the Tai Chi training or waitlist control, respectively.

### Secondary Outcomes

All the tests and measurements were completed at the baseline and post-intervention which were administered within 1 week before commencement and 1 week after completion of the Tai Chi training or waitlist control, respectively.

### Neuropsychological Tests

The Generalized Anxiety Disorder Scale (GAD-7) measures the level of anxiety of the participants [57]. It consists of seven self-report items with each scored between 0 and 3. The total GAD-7 score ranges from 0 to 21, with higher scores reflecting a higher level of anxiety. The Chinese version of GAD-7 showed satisfactory validity and reliability (Cronbach’s *α* = 0.89) [58].

The medical outcomes study 36-item short-form health survey (SF-36) measures the health-related quality of life of the participants. It consists of 36 self-reported items rated on different scales of scores ranging from 0 to 100. The items are grouped under the physical function, role limitation physical, bodily pain, general health, vitality, social function, role limitation emotional, and mental health subscales. Higher scores on each subscale reflect better subjective quality of life [59]. The Chinese version SF-36 was validated, and its Cronbach's α values for its subscales ranged from 0.72 to 0.88 [60].

### Salivary Cortisol Concentration

We adopted the 2015 cortisol arousal response guidelines [61] to collect the participants’ saliva samples. On each occasion, a saliva sample was collected three times within 45 min. The first collection was at time zero (time point 1), which was immediately after the participant got up from bed. The second and third time collections were at time 30 min after time zero (+ 30 min; time point (2)) and then 45 min (+ 45 min, time point (3)). The participant received three Salvette saliva tubes (Sarstedt, Italy) 1 day before the sample collection from the researcher assigned to conduct the outcome assessments. The instructions for the participant included not eating, drinking, brushing their teeth, smoking, or exercising until the sample collection was completed. The same procedure was repeated for the post-intervention test occasion. The cortisol concentration contained in the salivary samples was obtained using the salivary cortisol enzyme-linked immunosorbent assay kit (DRG Diagnostics, Germany).

### Structural Magnetic Resonance Imaging

Each participant completed the baseline and post-intervention MRI scans 1 week before commencement and 1 week after completion of the Tai Chi training or waitlist control, respectively. The scanning procedures were explained, and informed consent was obtained from the participants. MRI data acquisition covered T1-weighted high-resolution anatomical images using a Siemens Prisma 3.0 T. T1-weighted structural images were acquired with a three-dimensional magnetization-prepared rapid acquisition gradient-echo sequence, sagittal scanning (TR/TE/FOV = 2000 ms/1.73 ms/240 mm × 240 mm, flip angle = 15 degrees, layers = 160, layer thickness = 1 mm, and imaging matrix = 256 × 256). Data processing included preprocessing of the MRI data using the Statistical Parametric Mapping 12 (SPM 12) and the Computational Anatomy Toolbox 12 (CAT12) (http://dbm.neuro.uni-jena.de/cat12/) programs. High-dimensional Diffeomorphic Anatomical-Registration-Through-Exponentiated Lie-Algebra image registration was performed for nonlinear registration with the gray matter template of the standard Montreal Neurological Institute 152 space [62]. The normalized and segmented gray matter image was modulated and smoothed using isotropic Gaussian smoothing with a 10-mm full width at half maximum.

### Statistical Analyses

All data were analyzed by a statistician, who had not been involved in the delivery of the trial and collection of the data, using IBM Statistical Product Service Solutions (SPSS) v24.0 software. Independent sample t-test, nonparametric Mann–Whitney U-test, or Chi-square test were used to test possible between-group differences for the baseline measures. Linear mixed models (LMM) were performed to test the intervention effects with the Group × Time interaction, with *P* = 0.05 adjusted using the Holm–Bonferroni correction. The dependent variables were the primary and secondary outcome measure parameters.

For the salivary cortisol levels, we referred to the method proposed by Pruessner et al. to compute the area under the curve relative to the ground (AUCg), and the area under the curve with respect to increase (AUCi) to represent the cortisol dynamics changes across three time points within the first 45 min after waking from bed [63]. The AUCg is the total quantity of salivary cortisol measured at time zero, + 30 min, and + 45 min (see above). The AUCi is the “reactive” increase (or decrease) in the cortisol dynamics measured at the same three time points within 7 days after the participants completed the Tai Chi training. For the control group, the timelines of the cortisol dynamics measure matched with those of the Tai Chi training group. The Tai Chi training effects were tested with the Group × Time interaction effect using LMM. The ΔAUCg (post-minus-pre) of each participant was computed. The “pre” data were the baseline AUCg captured before commencing the Tai Chi training, while the “post” data were the post-intervention AUCg captured within 7 days after completing the training. The ΔAUCg of the participants was computed for the mediation analysis (see below).

For structural MRI, the ROIs selected to enter the analyses were the orbitofrontal cortex, insula, amygdala, caudate, putamen, globus pallidus, hippocampus, and thalamus. The selected ROIs were defined using the WFU_Pickatlas program and the Automated Anatomical Labeling template [64]. Individual and group ROI group differences were first examined by t-contrast with the participants’ age, sex, years of education, and total intracranial volume (TIV) entered as the covariates (SPM12 software). The mean values of the ΔGMV (post-minus-pre) and GMV (pre) and GMV (post) of the ROIs were extracted with Marsbar software (https://marsbartoolbox.github.io/). The statistical significance level of the analyses was set as the family-wise error (FWE) corrected at *P* < 0.05.

For the mediation analysis, Pearson’s or Spearman's correlation was performed to explore the relationships between variables that showed significant Group × Time effects. We used the PROCESS macro in SPSS v24.0 to estimate the possible mediating effects of ΔAUCg and/or ΔGMV on the Tai Chi training effect after controlling for the participants’ age, sex, years of education, and TIV. The general model set the group as the independent variable (experimental = 1 and control = 0), ΔPHQ-9 as the dependent variable, and ΔAUCg and/or ΔGMV as the mediators. All mediation analyses were based on ordinary least-squares regression and adopted a nonparametric bootstrapping procedure (5000 times), which gave rise to a bias-corrected confidence interval (CI) for the effect size inference. A *P* < 0.050 was considered statistically significant. In other words, significant effects were indicated if the 95% CI did not cover a zero value.

## Results

### Baseline Demographics and Outcome Measures

One hundred and twelve young adults met the inclusion criteria: 56 in each of the Tai Chi training and waitlist control groups (Additional file 1: Fig. S1). Nine participants dropped out during the study, resulting in 103 participants entering the final analyses. There were 49 participants in the Tai Chi training group and 54 participants in the waitlist control group. No significant between-group differences were revealed at the baseline in demographic characteristics such as age, sex, body mass index (BMI), TIV, and years of education (Table 1). There were no significant between-group differences in neuropsychological tests, daily activity time, and GMV of related regions in the reward circuit, but significant in AUCg and AUCi levels between groups at baseline comparison (Table 2 and Additional file 1: Tables S2–S3).

**Table 1:** Demographic and basic information of the participants

	Total (*n* = 103)	Experimental group (*n* = 49)	Control group (*n* = 54)	χ^2^/*t* /*Z*	*P*
*Gender*					
Female [n (%)]	70 (67.96%)	34 (69.39%)	36 (66.67%)	0.087	0.768
Male [n (%)]	33 (32.04)	15 (30.61)	18 (33.33)		
Age (years)^#^	19.00 (18.00, 20.00)	19.00 (19.00, 20.00)	19.00 (18.00, 20.00)	− 1.744	0.081
BMI^#^ (kg/m^2^)	20.90 (18.62, 23.24)	20.75 (18.95, 22.55)	20.81 (18.05, 23.99)	− 0.426	0.673
TIV (ml)	1451.39 (166.79)	1432.92 (118.78)	1455.30 (109.15)	− 1.539	0.127
Education (years)^#^	14.00 (13.00, 14.00)	13.00 (13.00,14.00)	14.00 (13.00, 14.00)	− 0.725	0.479
CES-D score^#^	20.00 (17.00, 27.00)	20.00 (18.00,27.00)	22.00 (16.50, 27.50)	− 0.589	0.558
PHQ-9 score^#^	9.00 (7.00, 12.00)	9.57 (4.11)	9.00 (6.00, 12.00)	− 0.799	0.427
GAD-7 score^#^	7.00 (4.00,10.00)	7.73 (4.65)	7.00 (4.00, 10.00)	− 0.670	0.506
SF-36 score	557.40 (96.98)	551.49 (91.18)	554.17 (503.34, 639.38)	− 0.587	0.558
AUCg^#^	4.72 (0.26, 8.08)	5.72 (3.73)	3.75 (0.22,7.70)	− 2.602	0.009
AUCi^#^	− 0.01 (− 0.15, 2.09)	0.46 (− 0.10, 2.82)	− 0.08 (− 0.19, 1.17)	− 2.239	0.025
Putamen GMV	0.30 (0.03)	0.30 (0.04)	0.29 (0.03)	0.971	0.335

*BMI* body mass index; *CES-D* Centre for Epidemiological Studies Depression Scale; *GAD-7* Generalized Anxiety Disorder Scale; *GMV* gray matter volume; *PHQ-9* Patient Health Questionnaire; *SF-36* the medical outcomes study 36-item short-form health survey; and *TIV* total intracranial volume

^#^Data are presented as median (P25, P75) due to an abnormal distribution and were examined using the Mann–Whitney U-test

**Table 2:** Summary of median scores and quartiles (in parentheses) and results of comparisons among groups and within group for the pre- and post-training assessments

Outcomes	Experimental group (*n* = 49)		Control group (*n* = 54)		Linear mixed model comparisons			Within-group comparisons (Post–Pre)	
	Pre	Post	Pre	Post	Group	Time	Group × Time	Experimental	Control
					*P*	*P*	*P*	*P*	*P*
PHQ-9^#^	9.57 (4.11)	5.00 (3.00,7.00)	9.00 (6.00,12.00)	8.00 (6.00,10.00)	0.001*	0.050*	0.002*	0.001*	0.012*
GAD-7^#^	7.73 (4.65)	3.00 (2.00,6.00)	7.00 (4.00,10.00)	6.00 (4.00,8.00)	0.017*	0.070	0.009*	0.001*	0.018*
SF-36^#^	551.49 (91.18)	648.75 (577.08,670.62)	554.17 (503.34,639.38)	575.66 (80.05)	0.025*	0.049*	0.002*	0.001*	0.053
AUCg^#^	5.72 (3.73)	4.05 (2.16,6.10)	3.75 (0.22,7.70)	3.39 (0.22,7.46)	0.398	0.956	0.010*	0.005*	0.785
AUCi^#^	0.46 (− 0.10,2.82)	1.01 (− 0.17,2.76)	− 0.08 (− 0.19,1.17)	− 0.08 (− 0.23,2.75)	0.732	0.084	0.343	0.737	0.739
Putamen GMV	0.30 (0.04)	0.29 (0.04)	0.29 (0.03)	0.30 (0.03)	0.581	0.233	< 0.001*	0.001*	0.234

The data are presented as ‾x ± sd or median (P25, P75) according to the data distribution; #Data were examined using the Mann–Whitney U-test due to the abnormal data distribution; *Indicates statistically significant differences

*AUCg* area under the curve relative to the ground; *AUCi* area under the curve with respect to increase; *GAD-7* Generalized Anxiety Disorder Scale; *GMV* gray matter volume; *PHQ-9* Patient Health Questionnaire; and *SF-36* the medical outcomes study 36-item short-form health survey

### Effects of Tai Chi Training on Primary and Secondary Outcome Measures

**Fig. 1:**
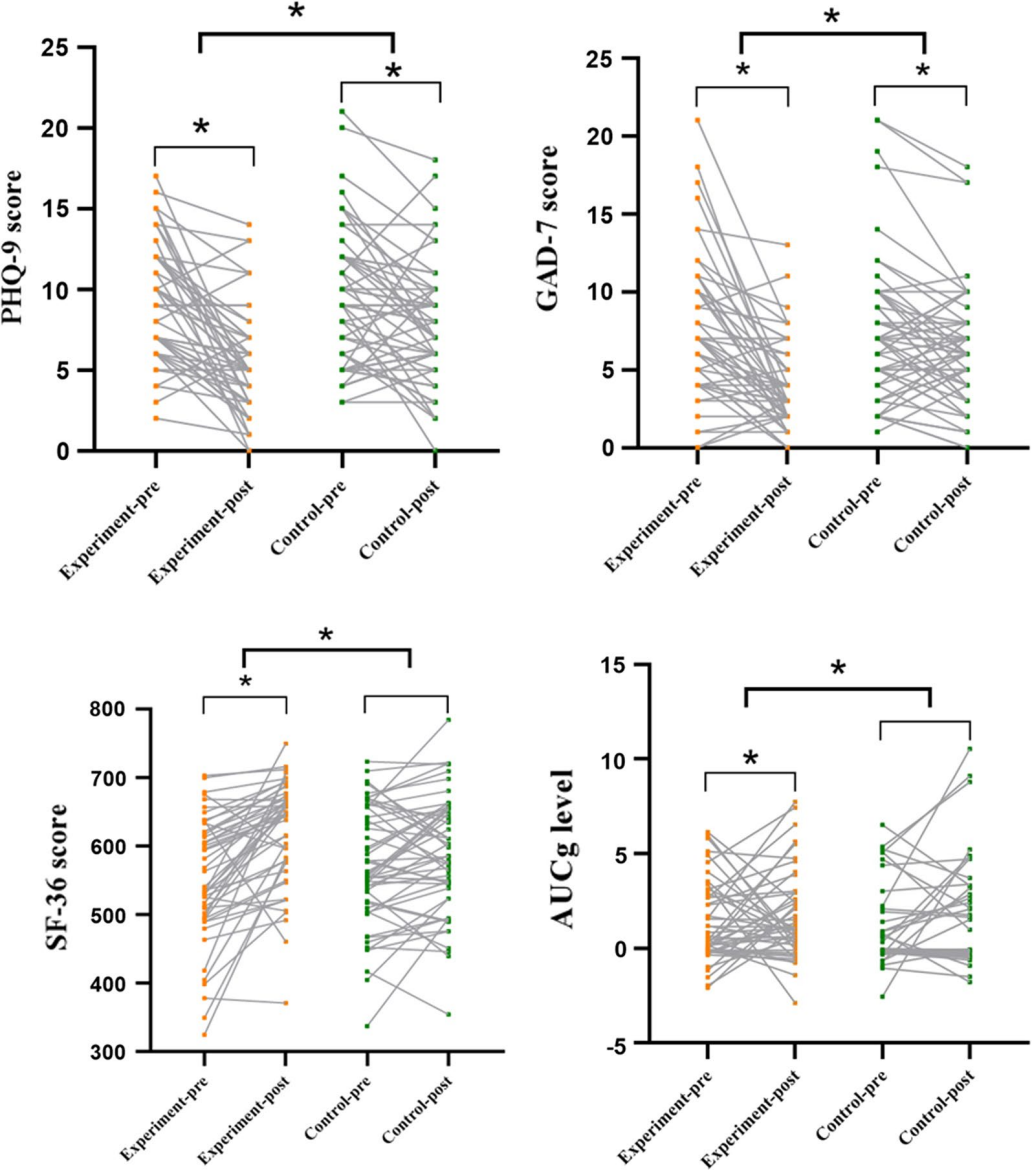
Top left: The PHQ-9 scores in the experimental group (*P* = 0.001 < 0.01) and in the control group (*P* = 0.012 < 0.05) were also significant. Upper right: The GAD-7 score decreased significantly after training in both the Tai Chi (*P* = 0.001 < 0.01) and the control group (*P* = 0.018 < 0.05). Bottom left: SF-36 in the experimental group increased significantly after training (*P* = 0.001 < 0.01) but was not significant in the control group (*P* = 0.053 > 0.05). Bottom right: The AUCg of the experimental group decreased significantly after training (*P* = 0.005 < 0.01) but it was not significant in the control group (*P* = 0.785 > 0.05). Each data point represents the value of a participant. Next to each arrow is the confidence interval (CI) for each effect. *P < 0.05. *Notes*: AUCg: area under the curve relative to the ground; GAD-7: Generalized Anxiety Disorder Scale; PHQ-9: Patient Health Questionnaire; and SF-36: the medical outcomes study 36-item short-form health survey

There were significant group, time, and Group × Time effects on the scores on the PHQ-9 score (*P* < 0.050) and SF-36 (*P* < 0.050). After 12 weeks, in the Tai Chi training group, there were significant decreases in the mean scores on the PHQ-9 (*P* = 0.001) and increases in the mean scores on the SF-36 (*P* = 0.001), while changes in the mean scores on the PHQ-9 and SF-36 were not statistically significant in the control group participants. Conversely, the GAD-7 scores showed significant Group × Time effects (*P* = 0.009), with both groups showing significant decreases in the post-intervention mean scores (*P* < 0.050) (Table 2 and Fig. 1). The AUCg and AUCi reflecting the cortisol dynamics showed significant Group × Time effects (Table 2 and Fig. 1). Within-group comparisons of the Tai Chi group participants showed that the post-intervention AUCg values were significantly lower than those at the baseline (see Table 2 and Fig. 1).

**Fig. 2:**
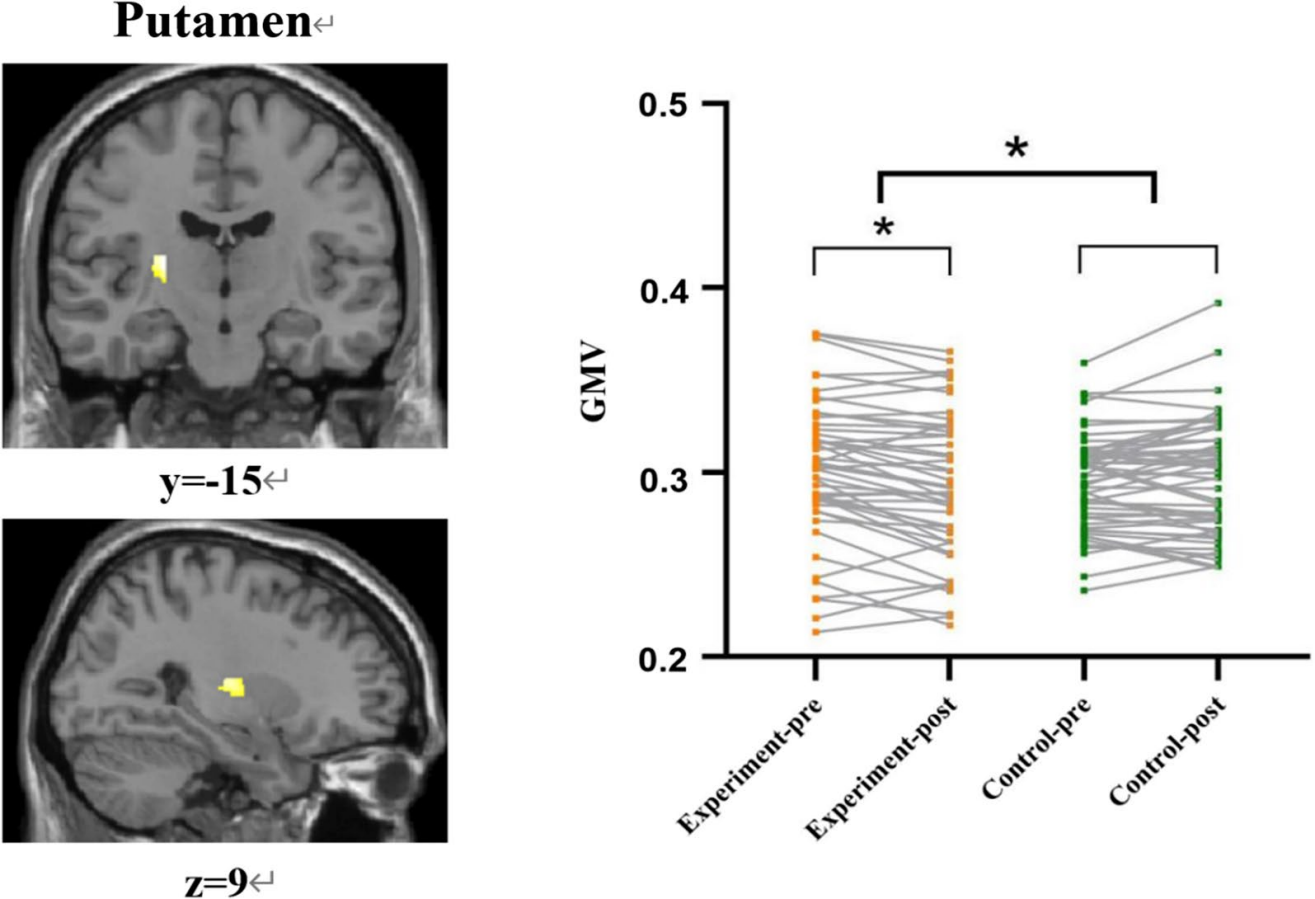
Left: Effects of Tai Chi training on putamen gray matter volume (GMV). There were significant differences within the group in the left putamen clusters after training (peak coordinates = − 26, − 15, 9, voxel number = 166, *P* = 0.020 < 0.05 after FWE correction). Right: The results of the linear mixed models (LMM) analysis in the region showed a significant group effect by the time interaction on the putamen GMV (*P* < 0.001). The putamen GMV in the experimental group decreased significantly after training (*P* = 0.001), but the change was not significant in the control group. Each data point represents the value of one participant. Next to each arrow is the confidence interval (CI) for each effect. *P < 0.05. *Notes*: GMV: gray matter volume

Among the eight ROIs, only the putamen showed significant changes in the GMV at the post-intervention measures (Additional file 1: Table S4). After controlling for age, sex, years of education, and TIV, there remained significant Group × Time effects (*P* < 0.001) on the left putamen’s gray matter volume (voxel number = 166, peak MNI: *x* = − 26, *y* = − 15, *z* = 9; *t* = 4.44, *P* = 0.020 after FWE correction) (Fig. 2; Additional file 1: Tables S2 and S4). Participants in the Tai Chi training group showed significant post-intervention decreases in the putamen GMV (*P* = 0.001), which compared to no significant differences in the waitlist control group (*P* = 0.232) (Table 2 and Fig. 2).

### Mediation Model for Tai Chi Training

**Fig. 3:**
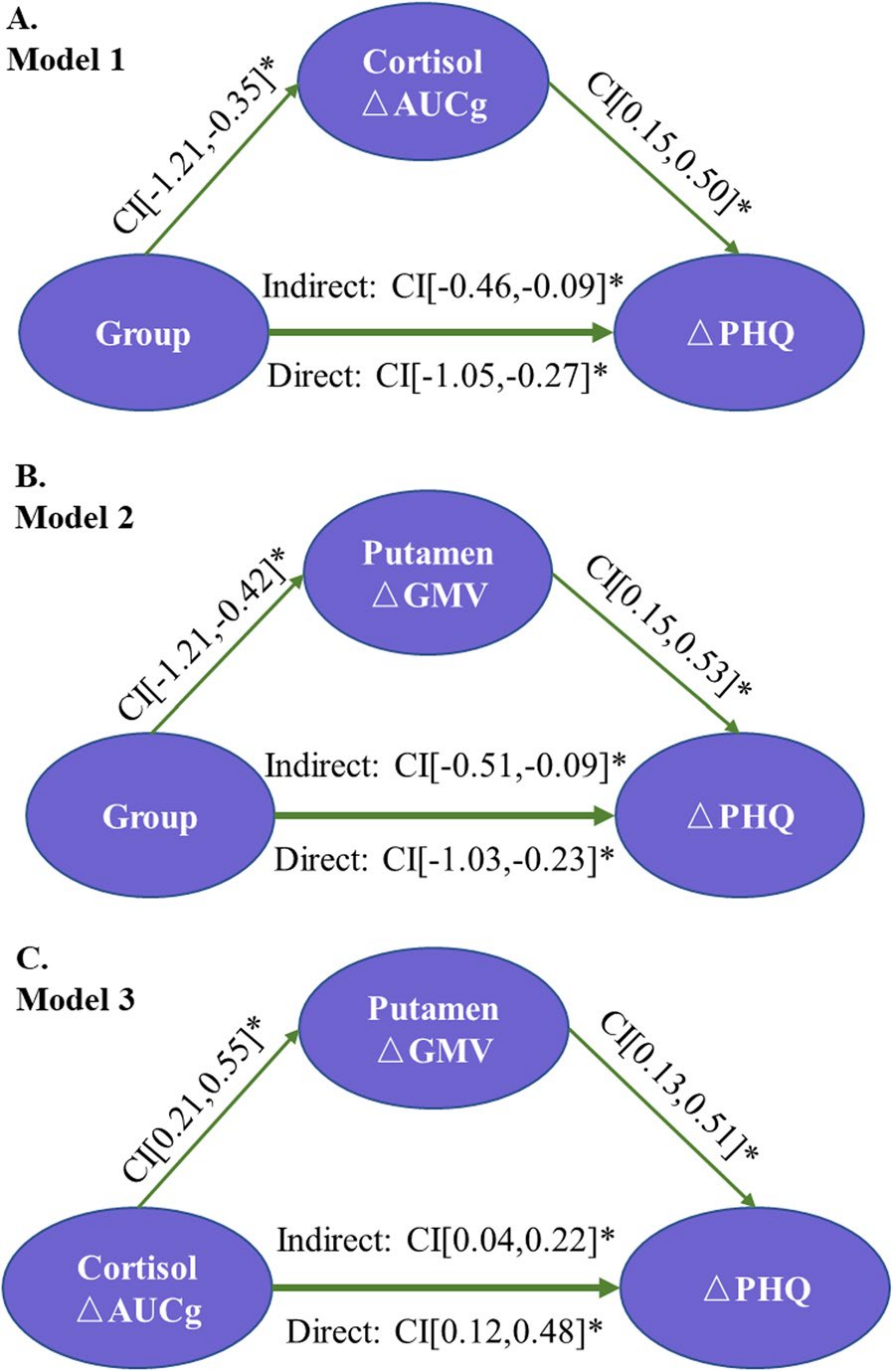
Diagram of the mediation model. **A** (Model 1): Group was the independent variable (experimental = 1 and control = 0). ΔAUCg was the mediator variable, and ΔPHQ was the dependent variable. **B** (Model 2): Group was the independent variable (experimental = 1 and control = 0). ΔGMV was the mediator variable, and ΔPHQ was the dependent variable. **C** (Model 3): ΔAUCg was the independent variable, ΔGMV was the mediator variable, and ΔPHQ was the dependent variable. *Notes*: AUCg: area under the curve relative to the ground; GMV: gray matter volume; and PHQ-9: Patient Health Questionnaire

Mediation models 1 and 2 were constructed, with group as the independent variable to test the relationship between the Tai Chi training effects and changes in the participants’ depressive symptoms, with the ΔPHQ scores as the dependent variable. Models 1 and 2 included ΔAUCg for the cortisol dynamics and ΔGMV of the putamen, respectively. Both models were found to be statistically significant, and the cortisol ΔAUCg (indirect CI [− 0.46, − 0.09]) and putamen ΔGMV (indirect CI [− 0.51, − 0.09]) were the mediators for the two-group ΔPHQ models (Fig. 3A and B).

Mediation model 3 was constructed to explore the differential roles of the cortisol ΔAUCg and putamen ΔGMV as the secondary treatment outcomes in modulating the participants’ ΔPHQ. Model 3 showed that cortisol ΔAUCg exerted direct effects on the ΔPHQ (direct CI [0.12, 0.48]), of which the effects were mediated by the putamen ΔGMV (indirect CI [0.04, 0.22]). Specifically, the cortisol ΔAUCg exerted positive effects on the putamen ΔGMV (bootstrap CI [0.21, 0.55]), and its effects were also positive on the ΔPHQ (bootstrap CI [0.13, 0.51]) (Fig. 3C).

**Fig. 4:**
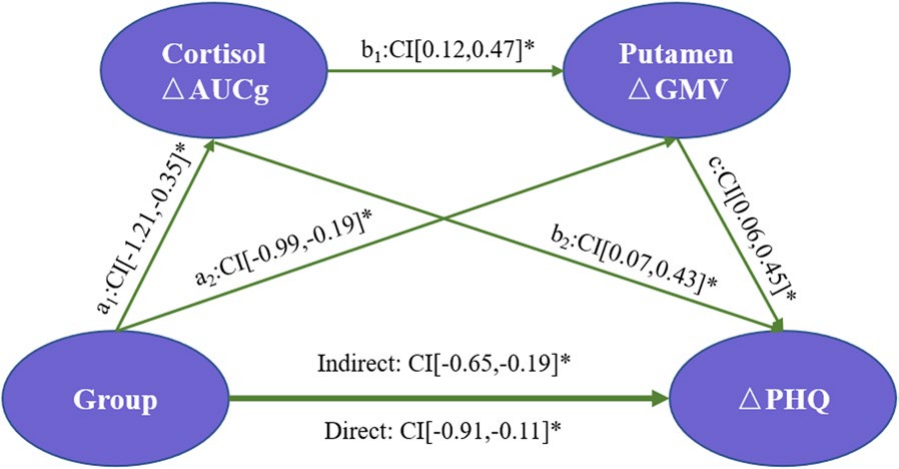
The serial mediation model showed that Tai Chi training affected ΔPHQ scores by modulating ΔAUCg and putamen ΔGMV after controlling age, sex, TIV, and years of education. This model demonstrated statistically significant total indirect effects, as well as indirect effects, through serial mediation. Group was the independent variable (experimental = 1 and control = 0); ΔGMV and ΔAUCg were the mediator variables; and ΔPHQ was the dependent variable. Age, sex, years of education, and TIV were covariates. *P < 0.05. *Notes*: AUCg: area under the curve relative to the ground; GMV: gray matter volume; and PHQ-9: Patient Health Questionnaire

The serial mediation model shown in Fig. 4 was further constructed to explore the differential roles of the cortisol ΔAUCg and putamen ΔGMV on the group ΔPHQ. The model showed that the effects of Tai Chi training were significantly mediated by both the ΔAUCg (a_1_: β = -0.78, CI [− 1.21, − 0.35]; b_2_: β = 0.25, CI [0.07, 0.43]) and putamen ΔGMV (a_2_: β = -0.59, CI [− 0.99, − 0.19], c: β = 0.25, CI [0.06, 0.45]). More importantly, this model showed that the partial mediation effect of the cortisol ΔAUCg on the ΔPHQ was through the putamen ΔGMV (b_1_: β = 0.29, CI[0.12, 0.47]) (Fig. 4; Additional file 1: Table S5).

## Discussion

This study revealed the 12-week Tai Chi training improved the depressive symptoms of a group of young participants with subthreshold depression. In addition to the symptoms, Tai Chi training was found to reduce the participants’ cortisol dynamics and decrease their putamen’s GMV. We further illustrated changes in the cortisol dynamics and putamen structure mediated the Tai Chi training effects on alleviating the depressive symptoms of the participants. Our findings suggested that the combined HPA axis activity and the reward circuitry add further evidence of the positive effects of Tai Chi training for young adult individuals with subthreshold depression.

The previous studies have reported the beneficial effects of Tai Chi as an intervention for alleviating the symptoms among adolescents with subthreshold depression [65] and older adults with depression [66]. The previous meta-analytic studies on mind–body exercises showed Tai Chi modulated HPA axis activity and lowered cortisol levels [67]. Higher levels of cortisol were found associated with neurotoxic effects [68, 69], while lower levels reduced damage to neurons and thus alleviated depressive symptoms [70]. The results of the present study are consistent with these findings. Tai Chi practice integrates slow and gentle movements and breathing techniques into psychocognitive functions (i.e., attention and imagination) [71], which relaxes the mind and body.

The previous studies have shown that Tai Chi training altered putamen volume in older adults [48]. The present study also found the participants in the Tai Chi training group showed decreases in the putamen’s volume which mediated the reduction in their depressive symptoms. The putamen is a component of the dorsal striatum in the reward circuitry and has been revealed to play a major role in the processing of reward information and reinforcement [72]. The putamen also mediates body movements, such as regulating motor planning and execution [73]. Tai Chi training requires planning, learning, and repetition of specific movements of the limbs and body, and their coordination is the essential ingredient to perform the Tai Chi style series [74]. One possible proposition is that the practice of the Tai Chi style could have been regarded as a reward to the participants, which activated the reward circuit. Such a proposition is challenged by the notion that Tai Chi training increased the putamen’s volume in older adults [48], which is opposite to our findings for the young adult participants. We further explore the pruning hypothesis [75] which may explain our findings. Different from brains in older adults studies revealed brains of adolescents and young adults undergoing rapid neurogenesis resulted in increased volumes [76, 77]. Synaptic pruning is a common development process among young brains [78, 79]. The putamen is a common site for synaptic pruning, and depression was reported to disrupt such a process [80, 81]. We speculate that the reduced putamen volume found in the Tai Chi training group could have been due to the post-intervention effect on restoring the pruning process [82]. Future study is to gather additional evidence to support our speculation.

The combined HPA axis activity and rewards mediating the Tai Chi training effects on depressive symptoms are a new finding. The association of the HPA axis and the reward circuitry has been found to link cortisol levels with motivation for rewards and performance [29]. The combined mechanism was proposed to involve inhibition of the adrenocorticotropin-releasing hormone receptors in the nucleus accumbens [83]. Elevated cortisol levels were reported to negatively regulate reward-related brain activity [84]. Conversely, lowered stress levels modulated the inhibitory striatal bed nucleus in the reward circuit [85]. The present study revealed a plausible combined HPA axis activity and reward circuitry effect associated with Tai Chi training, which is associated with improving depressive symptoms of the participants. Taken together, Tai Chi training might have downregulated the cortisol dynamics and upregulated the reward circuit activities. These effects could have elevated the motivation of the participants to engage in activities and, therefore, reduced depressive symptoms. Future studies should employ experimental designs to exert better controls on the activity engaged by the participants and closely monitor the changes in the HPA axis activity and the reward circuitry.

The current study has several limitations. Firstly, the single-blind design adopted in this study had a waitlist control group instead of an active control group. The active control group could have been participants involved in aerobic or stretching exercises. When compared with the waitlist group, participants in the Tai Chi training group could have developed a placebo effect because of the known intervention. The potential placebo effects such as increases in the level of motivation could have inflated the scores of the primary and data of the secondary outcomes and hence the treatment effects. Readers should be cautious when interpreting the findings revealed in the paper. Secondly, we did not include psychological traits such as interpersonal sensitivity and paranoia as controls that could have confounded the treatment effects. Future studies should validate our study findings by controlling the psychological traits and testing the effects of treatment in groups, including different age groups and types of depression. Thirdly, despite the use of the AUCg and AUCi as an attempt to normalize the participants’ cortisol levels, the varying cortisol levels across the participants and time of the day could have confounded the between- and within-group differences. The potential differences particularly those at the baseline are a limitation of the study informing the Tai Chi training effect. Future studies may consider capturing the changes in the cortisol dynamics a few days before and after the intervention to allow testing of the trends of changes.

## Conclusion

Twelve-week Tai Chi training was found to be effective in alleviating the symptoms of young participants with subthreshold depression. The symptom relief effect is likely to involve the downregulation of stress levels and the upregulation of reward circuit activity. These neural activities may improve the motivation of participants to engage in activities to combat depressive symptoms. We propose that the combined HPA axis and reward circuitry model are useful for explaining the positive Tai Chi training effect on depressive symptoms. Future study is to gather further evidence on the positive effects of mind–body exercises including Tai Chi with the combined HPA + RC model.

### Supplementary Information

**Additional file 1.** Supplementary Tables and Figure.

## Data Availability

The datasets used and/or analyzed during the current study are available from the corresponding author upon reasonable request.

## List of abbreviations

AUCg: Area under the curve relative to the ground
AUCi: Area under the curve with respect to increase
BMI: Body mass index
CES-D: Centre for Epidemiological Studies Depression Scale
CI: Confidence interval
GAD-7: Generalized Anxiety Disorder Scale
GMV: Gray matter volume
HPA: Hypothalamic–pituitary–adrenal
LMM: Linear mixed models
MDD: Major depressive disorder
PHQ-9: Patient Health Questionnaire
RC: Reward circuitry
SF-36: The medical outcomes study 36-item short-form health survey
TIV: Total intracranial volume

## References

[1] Daic GBD (2020). Global burden of 369 diseases and injuries in 204 countries and territories, 1990–2019: a systematic analysis for the Global Burden of Disease Study 2019. Lancet.

[2] Association AP, Force DT (2013). Diagnostic and statistical manual of mental disorders.

[3] Kroenke K (2017). When and how to treat subthreshold depression. JAMA.

[4] Zhang R, Peng X, Song X, Long J, Wang C, Zhang C (2022). The prevalence and risk of developing major depression among individuals with subthreshold depression in the general population. Psychol Med.

[5] Langer AI, Crockett MA, Bravo-Contreras M, Carrillo-Naipayan C, Chaura-Mario M, Gomez-Curumilla B (2022). Social and economic factors associated with subthreshold and major depressive episode in university students during the COVID-19 pandemic. Front Public Health.

[6] Aan HRM, Mathew SJ, Charney DS (2009). Neurobiological mechanisms in major depressive disorder. CMAJ.

[7] Tian H, Hu Z, Xu J, Wang C (2020). The molecular pathophysiology of depression and the new therapeutics. MedComm.

[8] Gonul AS, Akdeniz F, Taneli F, Donat O, Eker C, Vahip S (2005). Effect of treatment on serum brain-derived neurotrophic factor levels in depressed patients. Eur Arch Psychiatry Clin Neurosci.

[9] Rao U, Hammen CL, Poland RE (2009). Risk markers for depression in adolescents: sleep and HPA measures. Neuropsychopharmacology.

[10] Nguyen L, Kakeda S, Watanabe K, Katsuki A, Sugimoto K, Igata N (2020). Brain structural network alterations related to serum cortisol levels in drug-naive, first-episode major depressive disorder patients: a source-based morphometric study. Sci Rep.

[11] Zajkowska Z, Walsh A, Zonca V, Gullett N, Pedersen GA, Kieling C (2021). A systematic review of the association between biological markers and environmental stress risk factors for adolescent depression. J Psychiatr Res.

[12] Gaffrey MS, Barch DM, Bogdan R, Farris K, Petersen SE, Luby JL (2018). Amygdala reward reactivity mediates the association between preschool stress response and depression severity. Biol Psychiatry.

[13] Hoflich A, Michenthaler P, Kasper S, Lanzenberger R (2019). Circuit mechanisms of reward, anhedonia, and depression. Int J Neuropsychopharmacol.

[14] Yang X, Su Y, Yang F, Song Y, Yan J, Luo Y, Zeng J (2022). Neurofunctional mapping of reward anticipation and outcome for major depressive disorder: a voxel-based meta-analysis. Psychol Med.

[15] Admon R, Pizzagalli DA (2015). Dysfunctional reward processing in depression. Curr Opin Psychol.

[16] Weinstein AM (2023). Reward, motivation and brain imaging in human healthy participants—a narrative review. Front Behav Neurosci.

[17] Oh H, Lee J, Patriquin MA, Oldham J, Salas R (2023). Reward processing in psychiatric inpatients with depression. Biol Psychiatry Cogn Neurosci Neuroimaging.

[18] Vidal-Ribas P, Benson B, Vitale AD, Keren H, Harrewijn A, Fox NA (2019). Bidirectional associations between stress and reward processing in children and adolescents: a longitudinal neuroimaging study. Biol Psychiatry Cogn Neurosci Neuroimaging.

[19] Haber SN, Knutson B (2010). The reward circuit: linking primate anatomy and human imaging. Neuropsychopharmacology.

[20] Borsini A, Wallis A, Zunszain P, Pariante CM, Kempton MJ (2020). Characterizing anhedonia: a systematic review of neuroimaging across the subtypes of reward processing deficits in depression. Cogn Affect Behav Neurosci.

[21] Oei N, Both S, van Heemst D, van der Grond J (2014). Acute stress-induced cortisol elevations mediate reward system activity during subconscious processing of sexual stimuli. Psychoneuroendocrinology.

[22] Sesack SR, Grace AA (2010). Cortico-basal ganglia reward network: microcircuitry. Neuropsychopharmacology.

[23] Pan PM, Sato JR, Salum GA, Rohde LA, Gadelha A, Zugman A (2017). Ventral striatum functional connectivity as a predictor of adolescent depressive disorder in a longitudinal community-based sample. Am J Psychiatry.

[24] Bekhbat M, Li Z, Mehta ND, Treadway MT, Lucido MJ, Woolwine BJ (2022). Functional connectivity in reward circuitry and symptoms of anhedonia as therapeutic targets in depression with high inflammation: evidence from a dopamine challenge study. Mol Psychiatry.

[25] Huang W, Fang Y, Tan X, Zhao J (2022). Childhood trauma, stressful life events, and depression: exploring the mediating effect of cognitive flexibility. Psychol Trauma.

[26] Kim AW, Adam EK, Bechayda SA, Kuzawa CW (2020). Early life stress and HPA axis function independently predict adult depressive symptoms in metropolitan Cebu, Philippines. Am J Phys Anthropol.

[27] Menke A, Nitschke F, Hellmuth A, Helmel J, Wurst C, Stonawski S (2021). Stress impairs response to antidepressants via HPA axis and immune system activation. Brain Behav Immun.

[28] Peters AT, Van Meter A, Pruitt PJ, Briceno EM, Ryan KA, Hagan M (2016). Acute cortisol reactivity attenuates engagement of fronto-parietal and striatal regions during emotion processing in negative mood disorders. Psychoneuroendocrinology.

[29] Holloway AL, Schaid MD, Lerner TN (2023). Chronically dysregulated corticosterone impairs dopaminergic transmission in the dorsomedial striatum by sex-divergent mechanisms. Neuropsychopharmacology.

[30] Admon R, Treadway MT, Valeri L, Mehta M, Douglas S, Pizzagalli DA (2017). Distinct trajectories of cortisol response to prolonged acute stress are linked to affective responses and hippocampal gray matter volume in healthy females. J Neurosci.

[31] Hartling C, Fan Y, Weigand A, Trilla I, Gartner M, Bajbouj M (2019). Interaction of HPA axis genetics and early life stress shapes emotion recognition in healthy adults. Psychoneuroendocrinology.

[32] Langer K, Hagedorn B, Stock LM, Otto T, Wolf OT, Jentsch VL (2020). Acute stress improves the effectivity of cognitive emotion regulation in men. Sci Rep.

[33] Eikeseth FF, Denninghaus S, Cropley M, Witthoft M, Pawelzik M, Sutterlin S (2019). The cortisol awakening response at admission to hospital predicts depression severity after discharge in MDD patients. J Psychiatr Res.

[34] Chumbley JR, Hulme O, Kochli H, Russell E, Van Uum S, Pizzagalli DA, Fehr E (2014). Stress and reward: long term cortisol exposure predicts the strength of sexual preference. Physiol Behav.

[35] Lighthall NR, Sakaki M, Vasunilashorn S, Nga L, Somayajula S, Chen EY (2012). Gender differences in reward-related decision processing under stress. Soc Cogn Affect Neurosci.

[36] Cunningham S, Mazurka R, Wynne-Edwards KE, Milev RV, Pizzagalli DA, Kennedy S, Harkness KL (2021). Cortisol reactivity to stress predicts behavioral responsivity to reward moderation by sex, depression, and anhedonia. J Affect Disord.

[37] Liu X, Watanabe K, Kakeda S, Yoshimura R, Abe O, Ide S (2016). Relationship between white matter integrity and serum cortisol levels in drug-naive patients with major depressive disorder: diffusion tensor imaging study using tract-based spatial statistics. Br J Psychiatry.

[38] Admon R, Holsen LM, Aizley H, Remington A, Whitfield-Gabrieli S, Goldstein JM, Pizzagalli DA (2015). Striatal hypersensitivity during stress in remitted individuals with recurrent depression. Biol Psychiatry.

[39] Cramer H, Lauche R, Langhorst J, Dobos G (2013). Yoga for depression: a systematic review and meta-analysis. Depress Anxiety.

[40] Wang YY, Li XH, Zheng W, Xu ZY, Ng CH, Ungvari GS (2018). Mindfulness-based interventions for major depressive disorder: a comprehensive meta-analysis of randomized controlled trials. J Affect Disord.

[41] Zou L, Yeung A, Li C, Wei GX, Chen KW, Kinser PA (2018). Effects of meditative movements on major depressive disorder: a systematic review and meta-analysis of randomized controlled trials. J Clin Med.

[42] Zhang J, Gao T, Li Y, Song Z, Cui M, Wei Q (2023). The effect of Bafa Wubu of Tai Chi on college students’ anxiety and depression: a randomized, controlled pilot study. Front Physiol.

[43] Xu A, Zimmerman CS, Lazar SW, Ma Y, Kerr CE, Yeung A (2020). Distinct Insular functional connectivity changes related to mood and fatigue improvements in major depressive disorder following tai chi training: a pilot study. Front Integr Neurosci.

[44] Liu Z, Li L, Liu S, Sun Y, Li S, Yi M (2020). Reduced feelings of regret and enhanced fronto-striatal connectivity in elders with long-term Tai Chi experience. Soc Cogn Affect Neurosci.

[45] Kilpatrick LA, Siddarth P, Milillo MM, Krause-Sorio B, Ercoli L, Narr KL, Lavretsky H (2022). Impact of Tai Chi as an adjunct treatment on brain connectivity in geriatric depression. J Affect Disord.

[46] Alves PN, Foulon C, Karolis V, Bzdok D, Margulies DS, Volle E, Thiebaut DSM (2019). An improved neuroanatomical model of the default-mode network reconciles previous neuroimaging and neuropathological findings. Commun Biol.

[47] Liu J, Tao J, Liu W, Huang J, Xue X, Li M (2019). Different modulation effects of Tai Chi Chuan and Baduanjin on resting-state functional connectivity of the default mode network in older adults. Soc Cogn Affect Neurosci.

[48] Tao J, Liu J, Liu W, Huang J, Xue X, Chen X (2017). Tai Chi Chuan and Baduanjin increase grey matter volume in older adults: a brain imaging study. J Alzheimers Dis.

[49] Casey SM, Varela A, Marriott JP, Coleman CM, Harlow BL (2022). The influence of diagnosed mental health conditions and symptoms of depression and/or anxiety on suicide ideation, plan, and attempt among college students: findings from the Healthy Minds Study, 2018–2019. J Affect Disord.

[50] Slaughter-Acey J, Simone M, Hazzard VM, Arlinghaus KR, Neumark-Sztainer D (2023). More than identity: an intersectional approach to understanding mental-emotional well-being of emerging adults by centering lived experiences of marginalization. Am J Epidemiol.

[51] Radloff LS (1977). The CES-D scale: a self-report depression scale for research in the general population. Appl Psychol Meas.

[52] Sheehan DV, Lecrubier Y, Sheehan KH, Amorim P, Janavs J, Weiller E (1998). The mini-international neuropsychiatric interview (MINI): the development and validation of a structured diagnostic psychiatric interview for DSM-IV and ICD-10. J Clin Psychiatry.

[53] Xie X, Song J, Zhu J, Han M, He Y, Huang J (2021). The effectiveness of Tai Chi on the depressive symptom of young adults with subthreshold depression: a study protocol for a randomized controlled trial. Trials.

[54] China GAOS. 24-form tai chi chuan. 1999.

[55] Kroenke K, Spitzer RL, Williams JB (2001). The PHQ-9: validity of a brief depression severity measure. J Gen Intern Med.

[56] Wang W, Bian Q, Zhao Y, Li X, Wang W, Du J (2014). Reliability and validity of the Chinese version of the Patient Health Questionnaire (PHQ-9) in the general population. Gen Hosp Psychiatry.

[57] Spitzer RL, Kroenke K, Williams JB, Lowe B (2006). A brief measure for assessing generalized anxiety disorder: the GAD-7. Arch Intern Med.

[58] Tong X, An D, McGonigal A, Park SP, Zhou D (2016). Validation of the generalized anxiety disorder-7 (GAD-7) among Chinese people with epilepsy. Epilepsy Res.

[59] Ware JJ, Sherbourne CD (1992). The MOS 36-item short-form health survey (SF-36). I. Conceptual framework and item selection. Med Care.

[60] Li L, Wang HM, Shen Y (2003). Chinese SF-36 Health Survey: translation, cultural adaptation, validation, and normalisation. J Epidemiol Community Health.

[61] Stalder T, Kirschbaum C, Kudielka BM, Adam EK, Pruessner JC, Wust S (2016). Assessment of the cortisol awakening response: expert consensus guidelines. Psychoneuroendocrinology.

[62] Ashburner J (2007). A fast diffeomorphic image registration algorithm. Neuroimage.

[63] Pruessner JC, Kirschbaum C, Meinlschmid G, Hellhammer DH (2003). Two formulas for computation of the area under the curve represent measures of total hormone concentration versus time-dependent change. Psychoneuroendocrinology.

[64] Maldjian JA, Laurienti PJ, Kraft RA, Burdette JH (2003). An automated method for neuroanatomic and cytoarchitectonic atlas-based interrogation of fMRI data sets. Neuroimage.

[65] Zhang J, Qin S, Zhou Y, Meng L, Su H, Zhao S (2018). A randomized controlled trial of mindfulness-based Tai Chi Chuan for subthreshold depression adolescents. Neuropsychiatr Dis Treat.

[66] Lavretsky H, Milillo MM, Kilpatrick L, Grzenda A, Wu P, Nguyen SA (2022). A randomized controlled trial of Tai Chi Chih or health education for geriatric depression. Am J Geriatr Psychiatry.

[67] Liu X, Li R, Cui J, Liu F, Smith L, Chen X, Zhang D (2021). The effects of Tai Chi and Qigong exercise on psychological status in adolescents: a systematic review and meta-analysis. Front Psychol.

[68] Leonard BE (2018). Inflammation and depression: a causal or coincidental link to the pathophysiology?. Acta Neuropsychiatr.

[69] Soares JM, Sampaio A, Ferreira LM, Santos NC, Marques P, Marques F (2013). Stress impact on resting state brain networks. PLoS ONE.

[70] Tolahunase MR, Sagar R, Faiq M, Dada R (2018). Yoga- and meditation-based lifestyle intervention increases neuroplasticity and reduces severity of major depressive disorder: a randomized controlled trial. Restor Neurol Neurosci.

[71] Wang CC, Li K, Choudhury A, Gaylord S (2019). Trends in yoga, Tai Chi, and Qigong use among US adults, 2002–2017. Am J Public Health.

[72] Oh H, Lee J, Patriquin MA, Oldham J, Salas R (2021). Reward processing in psychiatric inpatients with depression. Biol Psychiatry Cogn Neurosci Neuroimaging.

[73] Monchi O, Petrides M, Strafella AP, Worsley KJ, Doyon J (2006). Functional role of the basal ganglia in the planning and execution of actions. Ann Neurol.

[74] Kong J, Wilson G, Park J, Pereira K, Walpole C, Yeung A (2019). Treating depression with Tai Chi: state of the art and future perspectives. Front Psychiatry.

[75] Keshavan M, Lizano P, Prasad K (2020). The synaptic pruning hypothesis of schizophrenia: promises and challenges. World Psychiatry.

[76] Wang Y, Zuo C, Xu Q, Hao L, Zhang Y (2021). Attention-deficit/hyperactivity disorder is characterized by a delay in subcortical maturation. Prog Neuropsychopharmacol Biol Psychiatry.

[77] Luby JL, Belden AC, Jackson JJ, Lessov-Schlaggar CN, Harms MP, Tillman R (2016). Early childhood depression and alterations in the trajectory of gray matter maturation in middle childhood and early adolescence. JAMA Psychiat.

[78] Cho RY, Walker CP, Polizzotto NR, Wozny TA, Fissell C, Chen CM, Lewis DA (2015). Development of sensory gamma oscillations and cross-frequency coupling from childhood to early adulthood. Cereb Cortex.

[79] Shaw P, Kabani NJ, Lerch JP, Eckstrand K, Lenroot R, Gogtay N (2008). Neurodevelopmental trajectories of the human cerebral cortex. J Neurosci.

[80] Zhang MM, Guo MX, Zhang QP, Chen XQ, Li NZ, Liu Q (2022). IL-1R/C3aR signaling regulates synaptic pruning in the prefrontal cortex of depression. Cell Biosci.

[81] Piochon C, Kano M, Hansel C (2016). LTD-like molecular pathways in developmental synaptic pruning. Nat Neurosci.

[82] Li C, Wang Y, Xing Y, Han J, Zhang Y, Zhang A (2022). Regulation of microglia phagocytosis and potential involvement of exercise. Front Cell Neurosci.

[83] Yuan Y, Wu W, Chen M, Cai F, Fan C, Shen W (2019). Reward inhibits paraventricular CRH neurons to relieve stress. Curr Biol.

[84] Montoya ER, Bos PA, Terburg D, Rosenberger LA, van Honk J (2014). Cortisol administration induces global down-regulation of the brain's reward circuitry. Psychoneuroendocrinology.

[85] Ulrich-Lai YM, Christiansen AM, Wang X, Song S, Herman JP (2016). Statistical modeling implicates neuroanatomical circuit mediating stress relief by 'comfort' food. Brain Struct Funct.

