# Supplementary material for: Effect of Tai Chi on Young Adults with Subthreshold Depression via a Stress–Reward Complex: A Randomized Controlled Trial
Source: Sports Med Open. 2023 Sep 28;9:90. doi: 10.1186/s40798-023-00637-w (PMC10539242; doi:10.1186/s40798-023-00637-w)
Supplement: Supplementary file 1 — Additional file 1. Supplementary Tables and Figure. [file 40798_2023_637_MOESM1_ESM.docx]

**Tai Chi training improves subthreshold depression in young adults via a stress-reward complex: A randomized controlled trial**

Jingsong Wu ^a,b,1^, Jian Song ^a,b,1^, Youze He ^a,b,1^, Zhaoying Li ^a^, Haiyin Deng ^a^, Zhenming Huang ^a^, Xiaoting Xie ^a^, Nichol M.L. Wong ^c,d^,

Jing Tao ^e,f, *^, Tatia M.C. Lee ^c,d*^, Chetwyn C.H. Chan ^g, *^

^a^ College of Rehabilitation Medicine, Fujian University of Traditional Chinese Medicine, Fuzhou, China.

^b^ The Academy of Rehabilitation Industry, Fujian University of Traditional Chinese Medicine, Fuzhou, China.

^c^ State Key Laboratory of Brain and Cognitive Sciences, The University of Hong Kong, Hong Kong

^d^ Laboratory of Neuropsychology & Human Neuroscience, The University of Hong Kong, Hong Kong

^e^ National-Local Joint Engineering Research Center of Rehabilitation Medicine Technology, Fujian University of Traditional Chinese Medicine, Fuzhou, China.

^f^ Fujian Key Laboratory of Rehabilitation Technology, Fujian University of Traditional Chinese Medicine, Fuzhou, China.

^g^ Department of Psychology, The Education University of Hong Kong, Hong Kong, China

^1^These authors contributed equally to this article

^*^ Correspondence:

Chetwyn C.H. Chan

Department of Psychology, The Education University of Hong Kong, Tai Po, Hong Kong, China.

E-mail: cchchan@eduhk.hk

Jing Tao, Ph.D.

Fujian University of Traditional Chinese Medicine

1 Huatuo Road, Minhou Shangjie

Fuzhou, Fujian 350122, P.R. China

Email: taojing01@fjtcm.edu.cn

Tatia M.C. Lee, Ph.D.

Rm 656, The Jockey Club Tower

The University of Hong Kong

Pokfulam Road, Hong Kong

E-mail: tmclee@hku.hk

**Methods**

**Study design and procedure**

This randomized controlled trial was conducted from December 2019 to December 2021 at Fujian University of Traditional Chinese Medicine in Fuzhou City, China. All eligible participants were randomly assigned in a 1:1 ratio to either the experimental group or the control group. All participants completed the neuropsychological assessments, brain structural imaging scans, and salivary cortisol collection before and after the 12-week intervention. Details of the research process were reported in the CONSORT flowchart. In this study, we first observed whether the tai chi intervention (detailed below) could improve the depressive symptoms of young adults with subthreshold depression. Then we examined how the benefits of tai chi training were mediated by changes in salivary cortisol levels and brain structure.

**Interventions**

The tai chi adopted in this study is the 24 simplified tai chi style that is recommended by the General Administration of Sport of China^1^. To guarantee the training quality, a coach with more than 5-year tai chi teaching experience was appointed to guide practices during the intervention. The tai chi training was carried out indoors in a gymnasium for 60 minutes each time, including 10 minutes of warm-up, 45 minutes of tai chi training, and 5 minutes of cooling down and it takes three times a week for 12 weeks. The details of tai chi training contents can be seen below in (supplementary table 1).

Participants in the control (waiting list) group maintained their daily lifestyle without intervention. All participants were told to keep a daily activity log during the study to observe any effect of regular physical activity. At the end of this study, participants were given the option of tai chi training according to their willingness.

**Supplemental Table 1. Content summary of the 24 simplified Tai Chi**

| [Movements of martial arts](javascript:;) | Name |
| --- | --- |
| 1 | Commencing form |
| 2 | Part the Wild Horse's Mane on Both Side |
| 3 | White Crane Spreads its Wings |
| 4 | Brush Knee and Twist Step on Both Side |
| 5 | Play Pipa |
| 6 | Repulse Monkey |
| 7 | Grasp the Bird's Tail (left) |
| 8 | Grasp the Bird's Tail (right) |
| 9 | Single Whip |
| 10 | Wave Hands Like Clouds |
| 11 | Single Whip |
| 12 | High Pat on Horse |
| 13 | Kick with Right Heel |
| 14 | Strike Opponent's Ears with Both Fists |
| 15 | Turn and Kick with Left Heel |
| 16 | Snake Creeps Down (left) |
| 17 | Snake Creeps Down (right) |
| 18 | Jade Lady Weaves Shuttles |
| 19 | Needle at Sea Bottom |
| 20 | Flash the Arm |
| 21 | Turn, Deflect Downward, Parry and Punch |
| 22 | Apparent Close up |
| 23 | Cross Hands |
| 24 | Closing Form |

**Information of daily activity logs**

Subjects in the experimental group and the control group recorded daily activity logs every week during the intervention period and summarized them into static activity time, low-intensity activity time, moderate-intensity activity time, and high-intensity activity time by referring to the physical activity outline compiled by Ainsworth BE et al^27^. Static work activities (0-1.5 METs): reading, learning, etc.; low-intensity activities (1.6-2.9 METs): household activities such as cleaning, walking, etc.; moderate intensity activities (3-5.9 METs): such as tai chi, yoga training, moderate pace brisk walking, stair climbing, etc.; Higher intensity activities (>6.0 METs): such as running, biking, dancing, etc.

According to the daily activity records, we found that during the intervention period, there was no significant difference in the average time of static work activities time, low-intensity activity time, and high-intensity activity time between the experimental group and the control group (*P*>0.05), the moderate-intensity activity time of experimental group was higher than that of the control group, and the difference was statistically significant (*P*<0.05). See supplementary table 2.

**Supplemental Table 2. Daily activity logs of the experimental group and the control group during the intervention period**

| Item | Experimental group (n=49) | Control group  (n=54) | Z | *P* |
| --- | --- | --- | --- | --- |
| Static work activities time (h)^#^ | 13.75(13.42,14.06) | 13.65(13.32,13.98) | -1.004 | 0.317 |
| Low-intensity activities time (h) ^#^ | 2.17(2.00,2.27) | 2.21(2.03,2.38) | -1.239 | 0.217 |
| Moderate intensity activities time (h) ^#^ | 1.01(0.56,1.42) | 0.55(0.50,1.13) | -3.018 | **0.002*** |
| Higher intensity activities time (h) ^#^ | 0.01(0.01,0.13) | 0.01(0.01,0.03) | -0.912 | 0.365 |

Notes: #: The data was present as Median (P25, P75) due to abnormal distribution and was examined by the Mann-Whitney U test.

**Sample size estimation**

In this randomized controlled trial, the Patient Health Questionnaire (PHQ-9) score was used as the main effect index. According to the results of a previous study^2^, 8-week mindfulness-based tai chi training had improved the PHQ-9 score of adolescents with subthreshold depression to 6.03 (SD 2.18), while the mean score of the PHQ-9 of participants was 8.10 (SD 2.52). The intervention effect value on PHQ-9 in the previous study was calculated by using Gpower 3.1.9.2 software (Effect size = 0.87), which was then used as the effect value in our study. The power value was set as 0.99 and the α value was 0.05. Hence, the estimated sample size was 100 in total, with 50 participants in each group. Considering the 10% drop-out rate, the final sample size needed for this study was 112, with 56 participants in each group.

[**Random**](javascript:;)**ized** [**allocation**](javascript:;) **and Blindness**

The randomized allocation sequence in this study was generated by an independent staff using SPSS24.0 in a ratio of 1:1 and all eligible participants were randomly assigned to the experimental group or control group. The random number range was preset to two codes A and B, which presented the group of experimental or control. Then these allocation results were printed and concealed in opaque, airtight envelopes. After baseline assessments, the result of allocation for each participant was told via telephone by this sequence manager, who was not involved in the enrolment, intervention, and outcome assessments. Moreover, the neuropsychology assessors and statisticians of this study were blinded to the group allocation and did not participate in the intervention process.

**Salivary cortisol sample collection and calculation**

In this study, to analyze the changes in salivary cortisol levels of participants with subthreshold depression, the salivary samples were collected before and after a 12-week intervention. Salvette saliva tubes (Sarstedt, Italy) were distributed to subjects before the day of collection. The researchers instructed the participants in detail about the methods and precautions of saliva collection. According to the 2015 International Society for PsychoneuroEndocrinology (ISPNE) cortisol arousal response expert consensus guidelines^3^, saliva samples were collected at three-time points: time point 1 (0 minutes after waking up), time point 2 (30 minutes after waking up) and time point 3 (45 minutes after waking up). Hence, participants in this study were asked to collect salivary samples at home or in their dormitory room at 0, 30, and 45 minutes after waking up in the morning, respectively. They were instructed not to eat, drink, brush their teeth, smoke, or exercise for 60 minutes after waking up and 30 minutes before saliva was collected. In addition, it was needed to chew the polyester swab from the saliva tube for at least 1 minute, then place the polyester swab into the inner tube of the saliva sampler. If oral diseases or inflammation happen, sample collection should be made after recovery.

The collected saliva sample tubes were temporarily stored in a refrigerator (4℃ temperature) before centrifugation and extraction. Then, these samples were recovered from dacron swabs and centrifuged at 3000 revolutions per minute for 15 min on the same day of collection. After centrifugation, the clean saliva could be obtained from the outer tubes and then removed into sterile EP tubes, frozen at -80℃ temperature. The process of salivary cortisol concentration was measured using the Salivary Cortisol ELISA Kit (DRG Diagnostics, Germany).

In this study, the area under the curve relative to ground (AUCg) and the area under the curve with respect to increase (AUCi) of cortisol arousal response were calculated according to Pruessner et al. 's formula^4^. AUCg reflects the total amount of cortisol secreted after awakening. AUCi reflects the dynamic change of cortisol after awakening. ΔAUCg (post-pre) and ΔAUCi (post-pre) were obtained by calculating the difference between AUCg and AUCi before and after intervention.

**Structural imaging data acquisition and processing**

The T1-weighted high-resolution anatomical images of participants were obtained by Siemens Prisma 3.0T NMR apparatus and 64-channel combined head and neck coil. T1-weighted structural images were acquired with the three-dimensional magnetization-prepared rapid acquisition gradient-echo sequence sagittal scanning, TR/TE/FOV=2000ms/1.73ms/240mm×240mm, flip angle=15 degrees, layers=160, layer thickness=1mm, imaging matrix=256×256. MRI scanning was performed at baseline and at the end of the intervention. The Statistical Parametric Mapping (SPM12, Institute of Neurology, London, UK) and the Computational Anatomy Toolbox (CAT12) (http://dbm.neuro.uni-jena.de/cat12/) software were applied for MRI data preprocessing. In CAT 12, images of nonlinear image registration were performed on T1 structural image using the high-dimensional Diffeomorphic-Anatomical-Registration-Through-Exponentiated-Lie-Algebra (DARTEL) image registration method, and then the image was registered with the gray matter template of the standard Montreal Neurological Institute (MNI) 152 space. The standardized image was segmented into Gray Matter (GM), White Matter (WM), and Cerebrospinal Fluid (CSF), and the segmented gray matter image was modulated so that the modulated image could be used for the comparison of brain gray matter. In the processing of spatially smooth, Isotropic Gaussian smoothing with Full Width at Half Maximum (FWHM) of 10mm×10mm×10mm was used for convolution processing so that data comparison could be carried out within a reasonable spatial scale.

**Results**

**Baseline demographics and information**

Given that 9 participants dropped out during the study (experimental group: 7 participants, control group: 2 participants), a total of 103 participants were included in the final analyses. Among them, 5 participants withdrew from this trial because of their graduation practice; 3 participants declined to participate again, and 1 participant was suspended due to a brain tumor. Hence, there were 49 participants in the tai chi group, and 54 participants in the control group. The flow chart of this study can be seen in the supplementary Figure 1.

**Supplementary Figure 1 Flow diagram of participants**

**
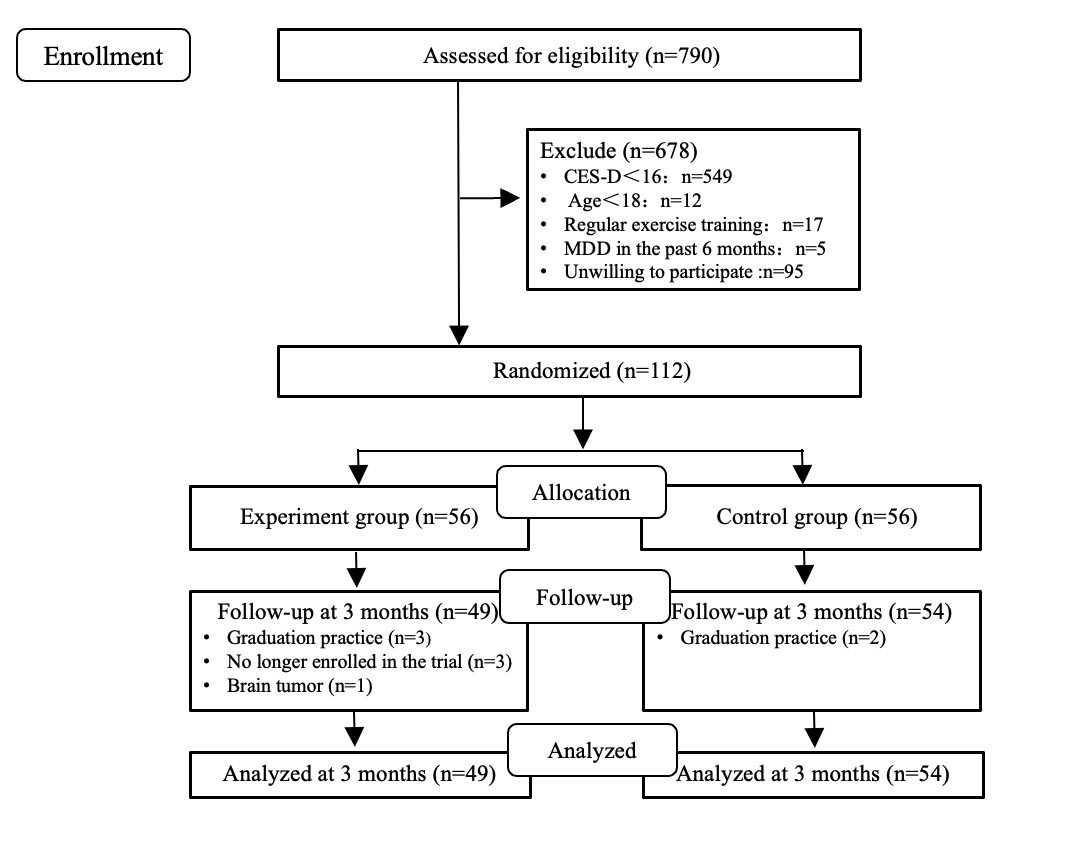
**

Evaluation of demographic characteristics showed that variables including age, gender, body mass index (BMI), intracranial volume (TIV), and years of education did not differ between the two groups (*P*> 0.05, see Table 1). In addition, there were no significant group differences in depression level (measured by the Centre for Epidemiological Studies Depression Scale), Patient Health Questionnaire (PHQ-9) score, Generalized Anxiety Disorder Scale (GAD-7) score, Medical Outcomes Study 36-Item Short-Form Health Survey (SF-36) score or gray matter volume (GMV) of predefined region of interest (ROIs) at baseline assessments (*P*> 0.05, see Table 1 and Supplementary Table3).

**Supplementary Table 3. Summary of baseline comparison on ROI’s GMV results**

|  | Total  (n=103) | Experimental group (n=49) | Control group  (n=54) | t | *P* |
| --- | --- | --- | --- | --- | --- |
| Putamen | 0.30  (0.03) | 0.30  (0.04) | 0.29  (0.03) | 0.971 | 0.335 |
| Amygdala | 0.54  （0.05） | 0.54  （0.05） | 0.55  （0.04） | -0.035 | 0.973 |
| Caudate | 0.36  （0.04） | 0.36  （0.04） | 0.36  （0.04） | 0.066 | 0.948 |
| Hippocampus | 0.40  （0.03） | 0.40  （0.04） | 0.41  （0.03） | -0.197 | 0.844 |
| Insula | 0.42  （0.04） | 0.42  （0.04） | 0.42  （0.04） | -0.516 | 0.607 |
| Orbitofrontal cortex | 0.39  （0.04） | 0.38  （0.04） | 0.39  （0.03） | -1.025 | 0.308 |
| Thalamus | 0.34  （0.03） | 0.34  （0.03） | 0.34  （0.03） | 0.345 | 0.731 |
| Globus pallidus | 0.40  （0.03） | 0.40  （0.03） | 0.40  （0.03） | -0.123 | 0.902 |

**Supplementary Table 4. Summary of comparison of ROI’s GMV results**

| Outcomes | Experimental group  (n=49) | | Control group  (n=54) | | Linear mixed model comparisons | | | Within-group comparisons  (Post - Pre) | |
| --- | --- | --- | --- | --- | --- | --- | --- | --- | --- |
|  | Pre | Post | Pre | Post | Group *P* | Time  *P* | Group×Time *P* | Experimental  *P* | Control  *P* |
| Putamen GMV | 0.24  (0.03) | 0.23  (0.03) | 0.23  (0.02) | 0.23  (0.03) | 0.581 | 0.233 | **<0.001*** | **0.001*** | 0.234 |
| Amygdala GMV | 0.54  (0.05) | 0.54  (0.05) | 0.55  (0.04) | 0.54  (0.04) | 0.961 | 0.415 | 0.956 | 0.343 | 0.455 |
| Caudate GMV | 0.36  (0.04) | 0.35  (0.03) | 0.36  (0.04) | 0.36  (0.03) | 0.975 | 0.023* | 0.775 | 0.088 | **0.020*** |
| Hippocampus GMV | 0.40  (0.04) | 0.40  (0.04) | 0.41  (0.03) | 0.40  (0.03) | 0.816 | 0.459 | 0.808 | 0.256 | 0.493 |
| Insula GMV | 0.42  (0.04) | 0.42  (0.04) | 0.42  (0.04) | 0.42  (0.04) | 0.641 | 0.228 | 0.833 | 0.413 | 0.211 |
| Orbitofrontal cortex GMV | 0.38  (0.04) | 0.38  (0.04) | 0.39  (0.03) | 0.39  (0.03) | 0.324 | 0.032* | 0.856 | **0.001*** | **0.012*** |
| Thalamus GMV | 0.34  (0.03) | 0.33  (0.03) | 0.34  (0.03) | 0.33  (0.03) | 0.994 | 0.311 | 0.166 | **0.012*** | 0.261 |
| Globus pallidus GMV | 0.40  (0.03) | 0.40  (0.03) | 0.40  (0.03) | 0.40  (0.03) | 0.633 | 0.820 | 0.252 | 0.197 | 0.811 |

Notes:*: The difference was statistically significant.

The LMM results showed a significant group × time interaction effect of putamen GMV (*P*<0.001) but no significant interaction effect on other ROI’s GMV (See Table 3 and Supplementary Table 4).

**Supplementary Table 5. Summary of median scores and quartiles (in parentheses) and ITT results of comparisons among groups**

| Outcomes | Experimental group (n=49) | | Control group (n=54) | | Linear mixed model comparisons | | |
| --- | --- | --- | --- | --- | --- | --- | --- |
|  | Pre | Post | Pre | Post | Group | Time | Group×Time |
|  |  |  |  |  | *P* | *P* | *P* |
| PHQ-9^#^ | 8.57  (7.00,12.00) | 5.00  (3.00,8.16) | 9.00  (5.75,12.00) | 7.50  (5.75,10.00) | **0.002*** | **0.049*** | **0.002*** |
| GAD-7^#^ | 7.00  (5.00,10.00) | 4.00  (2.00,6.42) | 7.00  (4.00,10.00) | 6.00  (4.00,8.00) | **0.017*** | 0.070 | **0.009*** |
| SF-36^#^ | 579.44  (502.08,616.25) | 642.50  (578.75,666.25) | 558.75  (509.58,656.25) | 583.75  (522.92,652.50) | **0.025*** | **0.049*** | **0.002*** |
| AUCg  (Time 1-3)^#^ | 5.06(3.18,8.31) | 4.28(2.82,5.98) | 0.33(0.22,7.24) | 0.36(0.21,7.24) | 0.398 | 0.956 | **0.010*** |
| AUCi  (Time 1-3)^#^ | 0.85  (-0.07,2.68) | 1.09  (-0.03,2.44) | -0.08  (-0.18,0.92) | -0.09  (-0.21,2.43) | 0.732 | 0.084 | 0.343 |

Notes: [1] The data are presented as ‾x±sd or median (P25, P75) according to the data distribution; [2]#: Data were examined using the Mann-Whitney U test due to the abnormal data distribution; [3]*: Indicates statistically significant differences. AUCg: Area Under the curve relative to the ground; AUCi: Area Under the curve with respect to increase; GAD-7: Generalized Anxiety Disorder Scale; GMV: Gray matter volume; PHQ-9: Patient Health Questionnaire; SF-36: The Medical Outcomes Study 36-Item Short-Form Health Survey.

**ΔAUCg and ΔGMV of putamen serially mediated the intervention effect of Tai Chi**

The results of Pearson correlation analysis showed that ΔPHQ was significantly correlated with ΔAUCg (*r*=0.329, *P*=0.001) and putamen ΔGMV (*r*=0.253, *P*=0.010). And the putamen ΔGMV was also significantly correlated with Group (*r*=-0.398, *P*<0.001) and ΔAUCg (*r*=0.206, *P*=0.037). The ΔAUCg was correlated with Group (*r*=-0.260, *P*=0.008). However, there was no significant association found among the changes in GAD-7 scores, SF-36 scores, ΔAUCg, ΔAUCi, and ΔGMV. Hence, this study further investigated possible relationships among ΔPHQ, ΔAUCg, and putamen ΔGMV by conducting a serial mediation analyses model on PHQ scores.

In model 1 mediation analysis, tai chi had a direct effect on ΔPHQ score (*P*<0.05) and was also partially mediated by the ΔAUCg (indirect CI: [-0.46, -0.09]). Specifically, the tai chi group had a negative effect on ΔAUCg (Bootstrap CI: [-1.21, -0.35]), and ΔAUCg also had a positive effect on ΔPHQ score (Bootstrap CI: [0.15, 0.50]) (See Fig.3).

In model 2 mediation analysis, tai chi had a direct effect on Δ PHQ score (*P*<0.05) and was partially mediated by the putamen ΔGMV (indirect CI: [-0.51, -0.09]). Specifically, the tai chi group had a negative effect on putamen ΔGMV (Bootstrap CI: [-1.21, -0.42]) and ΔGMV also had a positive effect on ΔPHQ score (Bootstrap CI: [0.15,0.53]) (See Fig.3).

In model 3 mediation analysis, ΔAUCg directly affected the ΔPHQ (direct CI: [0.12, 0.48]) and partially mediated by putamen ΔGMV (indirect CI: [0.04, 0.22]). Specially, ΔAUCg had a positive effect on putamen ΔGMV (Bootstrap CI: [0.21, 0.55]), which also had a positive effect on the ΔPHQ (Bootstrap CI: [0.13, 0.51]) (See Fig.3).

In the serial mediation analysis, the results identified the significant mediating effect of ΔAUCg and putamen ΔGMV on the ΔPHQ (direct CI: [-0.91, -0.11]; indirect CI: [-0.65, -0.19]). Specifically, the intervention effect of tai chi was significantly mediated by both ΔAUCg (a_1_: β=-0.78, CI[-1.21,-0.35]; b_2_: β=0.25, CI[0.07, 0.43]) and putamen ΔGMV (a_2_: β=-0.59, CI[-0.99,-0.19], c: β=0.25, CI[0.06, 0.45]). More importantly, this model showed that the partial mediation effect of ΔAUCg was via modulating the putamen ΔGMV (b_1_: β=0.29, CI[0.12, 0.47]). (See Fig.4 and Supplementary Table 5)

**Supplementary Table 6. Path coefficients from the serial mediation model**

| Model pathways | β | *P* | Indirect effect [95% CI] |
| --- | --- | --- | --- |
| a_1_ | -0.78 | **0.004*** | [-1.21,-0.35]* |
| a_2_ | -0.59 | **0.004*** | [-0.99,-0.19]* |
| b_1_ | 0.29 | **0.001*** | [0.12,0.47] |
| b_2_ | 0.25 | **0.006*** | [0.07,0.43]* |
| c | 0.25 | **0.010*** | [0.06,0.45]* |
| d |  |  | -0.59[-0.91,-0.11]* |
| a_1_*b_2_ |  |  | -0.20[-0.37,-0.06]* |
| a_2_*c |  |  | -0.15[-0.29,-0.03]* |
| a_1_*b_1_*c |  |  | -0.06[-0.15,-0.01]* |
| dd |  |  | -0.40[-0.66,-0.19]* |

Notes: a_1_: the effect of group on ΔAUCg; a_2_: the effect of group on putamen ΔGMV; b_1_: the effect of ΔAUCg on putamen ΔGMV; b_2_: the effect of ΔAUCg on ΔPHQ; c: the effect of putamen ΔGMV on ΔPHQ; d: the direct effect of group on ΔPHQ; a_1_*b_2_: the partial mediating effect of ΔAUCg on group and ΔPHQ; a_2_*c: the partial mediating effect of putamen ΔGMV on group and ΔPHQ; a_1_*b_1_*c: the serial mediating effect of ΔAUCg and putamen ΔGMV on group and ΔPHQ; dd: the indirect effect of group on ΔPHQ.

**Abbreviations**

AAL: Automated Anatomical Labelling; AUCg: Area Under the Curve relative to ground; AUCi: Area Under the Curve with respect to increase; BMI: Body Mass Index; CAT 12: Computational Anatomy Toolbox 12; CES-D: Centre for Epidemiological Studies Depression Scale; DARTEL: Diffeomorphic Anatomical Registration Through Exponentiated Lie Algebra; DSM-V: Diagnostic and Statistical Manual of Mental Disorders-V; ELISA: Enzyme Linked Immunosorbent Assay; FWE: Family-Wise Error; GAD-7: Generalized Anxiety Disorder Scale; GMV: Gray Matter Volume; HPA: Hypothalamic-Pituitary-Adrenal; MAAS: Mindful Attention and Awareness Scale; MDD: Major Depressive Disorder; MINI: Mini International Neuropsychiatric Interview; MNI: Montreal Neurological Institute; MRI: magnetic resonance image; PHQ-9: Patient Health Questionnaire; PSS: Perceived Stress Scale; ROI: Region of Interest; SAS: Statistical Analysis System; SF-36: Medical Outcomes Study 36-Item Short-Form Health Survey; SPM 12: Statistical Parametric Mapping 12; SPSS: Statistical Product Service Solutions software; TIV: Intracranial Volume; WFU: Wake Forest University.

**REFERENCES**

1 China GAOS. 24-form tai chi chuan.; 1999.

2 Zhang J, Qin S, Zhou Y*, et al.*. A randomized controlled trial of mindfulness-based tai chi chuan for subthreshold depression adolescents. *Neuropsych Dis Treat* 2018;14:2313-2321.

3 Stalder T, Kirschbaum C, Kudielka BM*, et al.*. Assessment of the cortisol awakening response: expert consensus guidelines. *Psychoneuroendocrinology* 2016;63:414-32.

4 Pruessner M, Hellhammer DH, Pruessner JC*, et al.*. Self-reported depressive symptoms and stress levels in healthy young men: associations with the cortisol response to awakening. *Psychosom Med* 2003;65(1):92-99.
